# Supplementary material for: Elevated Production of Mitochondrial Reactive Oxygen Species via Hyperthermia Enhanced Cytotoxic Effect of Doxorubicin in Human Breast Cancer Cell Lines MDA-MB-453 and MCF-7
Source: Int J Mol Sci. 2020 Dec 15;21(24):9522. doi: 10.3390/ijms21249522 (PMC7765207; doi:10.3390/ijms21249522)
Supplement: Supplementary file 1 [file ijms-21-09522-s001.pdf]

International Journal of
Molecular Sciences

MDPI

Supplementary Figure

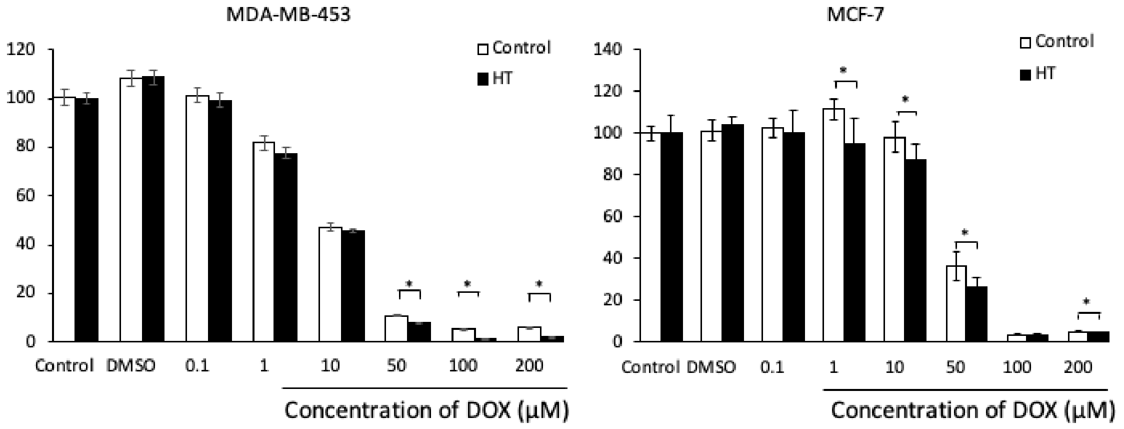

Figure S1. MDA-MB-453 and MCF-7 cells were treated with or without HT and cell viabilities after DOX treatment were measured using the CCK-8 method. Statistical significance was tested by Student's t-test. $n = 4$ (MDA-MB-453), $n = 6$ (MCF-7), error bar; S.D. *$p < 0.01$.
